# Antipathogenic Compounds That Are Effective at Very Low Concentrations and Have Both Antibiofilm and Antivirulence Effects against Pseudomonas aeruginosa

**DOI:** 10.1128/Spectrum.00249-21

**Published:** 2021-09-08

**Authors:** Hyeon-Ji Hwang, Heejeong Choi, Sojeong Hong, Hyung Ryong Moon, Joon-Hee Lee

**Affiliations:** a Department of Pharmacy, College of Pharmacy, Pusan National University, Busan, South Korea; b Department of Manufacturing Pharmacy, College of Pharmacy, Pusan National University, Busan, South Korea; University of Maryland School of Pharmacy

**Keywords:** *Pseudomonas aeruginosa*, quorum sensing, biofilm, virulence, antivirulence, antibiofilm, chemical screening, antipathogenic compound

## Abstract

Pseudomonas aeruginosa, a human pathogen, causes both acute and chronic infections that are mediated by virulence factor production and biofilm formation. Since both characteristics of P. aeruginosa are regulated by quorum sensing (QS), we screened 126 synthetic chemicals for anti-QS activity and finally selected the compounds that have both antivirulence and antibiofilm activities. To efficiently screen the chemical library, the following reporter-based bioassay systems were used: the QS- or biofilm-specific promoter-*lacZ* fusions (*lasI*_p_- or *PA1897*_p_-*lacZ* for the QS activity and *cdrA*_p_-*lacZ* for measuring the intracellular c-di-GMP levels). We also measured the production of virulence factors and biofilm formation in P. aeruginosa. A small-animal infection model using mealworms was also used for virulence analysis. From this screening, MHY1383 and MHY1387 were found to have both antivirulence and antibiofilm activities in P. aeruginosa. Most importantly, MHY1383 and MHY1387 exhibited these activities at very low concentrations, showing a significant anti-QS effect at 100 pM and an antibiofilm effect at 1 to 10 pM. By treating P. aeruginosa with these compounds, the virulence factor production and biofilm formation of P. aeruginosa were significantly reduced. These compounds can be developed as promising antipathogenic and antibiofilm drugs that can be applied in situations where such compounds must be used in an extremely low concentration. Our findings also offer a significant advantage for developing therapeutic agents with few adverse side effects.

**IMPORTANCE** Many antibiotics are increasingly losing their efficacy due to antibiotic resistance mediated by biofilm formation. In this study, we screened a synthetic chemical library and discovered several compounds that have both antivirulence and antibiofilm effects against Pseudomonas aeruginosa, a notorious human pathogen. Two of them had these effects at extremely low concentrations and are expected not to develop resistance, unlike conventional antibiotics, because they have no effect on the growth of bacteria. Our results strongly suggest that these compounds act on the target in a noncompetitive manner, indicating that they are distinct from other previously known quorum sensing inhibitors or biofilm inhibitors. Our findings offer a significant advantage for developing therapeutic agents with few adverse side effects.

## INTRODUCTION

In many pathogenic bacteria, the expression of virulence factors is mainly controlled by the quorum sensing (QS) system that regulates a large number of genes in a cell density-dependent manner (1). The QS-controlled physiology includes the production of virulence factors, sporulation, and biofilm formation, while QS-based cell to cell signaling allows bacteria to exhibit a highly coordinated and cooperative group behavior, typically, biofilm formation (2). The biofilm is a very protective life mode; hence, most antibiotics are not effective to the cells in biofilms, and the infections mediated by biofilm formation often become chronic even with antibiotic medication (3). Therefore, most infectious diseases are caused by the pathogens that have a QS and biofilm formation capability, and there is a need for substances capable of effectively antagonizing these two activities of pathogenic bacteria.

Bacterial infections can be either acute or chronic. Acute infections are usually caused by the virulence factors produced by pathogens, while chronic infections are mostly caused by biofilms of pathogens that hyperactivate host immune factors to damage the infection sites; the transition between these two modes of infection has been suggested to be inversely regulated (4–7). Therefore, substances that can inhibit both of these two modes simultaneously can be of great help in controlling infection.

Pseudomonas aeruginosa, an opportunistic human pathogen, also produces a large arsenal of virulence factors and forms robust biofilms through QS regulation, so it is very difficult to treat once an infection occurs (1, 8). P. aeruginosa has two major QS signals, acyl homoserine lactones (acyl-HSLs) and 2-alkyl-4-quinolones (AQs). The major acyl-HSLs P. aeruginosa produces are N-3-oxododecanoyl-HSL (3OC12-HSL) and N-butyryl-l-HSL (C4-HSL), which bind to and activate their cognate receptors, LasR (for 3OC12-HSL), QscR (for 3OC12-HSL), and RhlR (for C4-HSL) (1, 9). Meanwhile, two AQs, 3,4-dihydroxy-2-heptylquinoline (PQS, Pseudomonas quinolone signal) and its precursor 4-hydroxy-2-heptylquinoline (HHQ), bind to and activate PqsR (10). In the QS response, as cell density increases, the QS signal molecules accumulate to a certain threshold concentration and then bind to their specific receptors to activate the expression of target genes (1). In P. aeruginosa, one of these targets is the set of genes encoding extracellular proteases, which are representative virulence factors (11, 12). Biofilm formation by P. aeruginosa has also been reported to be dependent on QS (13). Hence, it has been suggested that bacterial virulence for both acute and chronic infections might be controlled by QS signaling, and researchers have proposed the use of several strategies in order to modulate it—QS inhibitors that can outcompete QS signals from their receptors, QS signal quenchers that can degrade or sequester QS signals, and novel xeno- or allosignals that can modulate the QS signaling of specific bacteria (14). Based on these strategies, we screened the anti-QS compounds that can attenuate P. aeruginosa virulence and biofilm formation (15–17).

As an extension of this effort, we explored novel anti-QS compounds from a synthetic chemical library. In particular, in order to obtain better materials compared to previous ones, we tried to find a compound that has the ability to inhibit both virulence and biofilm formation with no effect on growth and that can act even at very low concentrations. Unlike conventional antibiotics, the QS inhibitors that have no effect on growth presumably allow resistance to develop less frequently (18). Substances that are effective even at extremely low concentrations are not only advantageous in combination with other antibiotics, but also have the great advantage of reducing toxicity. It is also very advantageous for application to the environment or industrial facilities. In this study, we screened novel synthetic compounds (MHY series) and discovered some promising compounds, MHY1383 and 1387. They have both antivirulence and antibiofilm activities against P. aeruginosa and, most importantly, exhibited their effects at extremely low concentrations.

## RESULTS

### Four MHYs inhibited QS and virulence factor production of P. aeruginosa.

**FIG 1:**
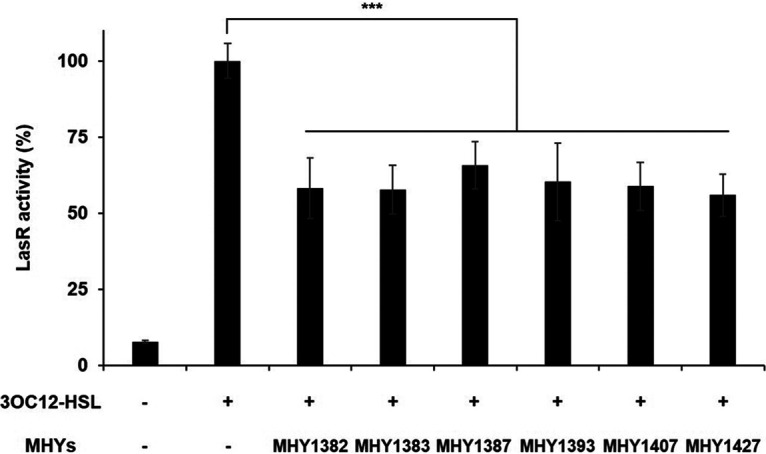
Several MHYs inhibited the QS response in P. aeruginosa. We screened 126 synthetic compounds (MHYs) for anti-LasR activity by using the E. coli dual plasmid reporter harboring pSC11 and pJN105L. 3OC12-HSL and MHYs were used at the concentrations of 50 nM and 5 μM, respectively, and LasR expression was induced by adding 0.4% arabinose. The activities were presented relative to the sample with only 3OC12-HSL (which corresponds to 100%). ***, *P < *0.005.

MHY compounds in the chemical library were screened for anti-QS activity by the reporter-based bioassay. This bioassay was performed with the Escherichia coli dual plasmid reporter harboring pSC11 (*lasI*_p_-*lacZ* fusion plasmid) and pJN105L (LasR-expressing plasmid), which was designed for measuring the activity of LasR, the master QS regulator of P. aeruginosa (16). This reporter shows a very high β-galactosidase activity when 3OC12-HSL is added but shows a reduced activity when an effective LasR inhibitor is additionally added. To better see the competition between 3OC12-HSL and inhibitors to LasR, 3OC12-HSL was added at the concentration for the half-maximal activation of LasR (50 nM), and the inhibitors were added at 5 μM, a 100-times-higher concentration than that of 3OC12-HSL. Under these conditions, we screened 126 MHYs, and 6 MHYs (MHY1382, MHY1383, MHY1387, MHY1393, MHY1407, and MHY1427) significantly inhibited the activity of LasR (Fig. 1). They reduced the LasR activity by more than 35%.

**FIG 2:**
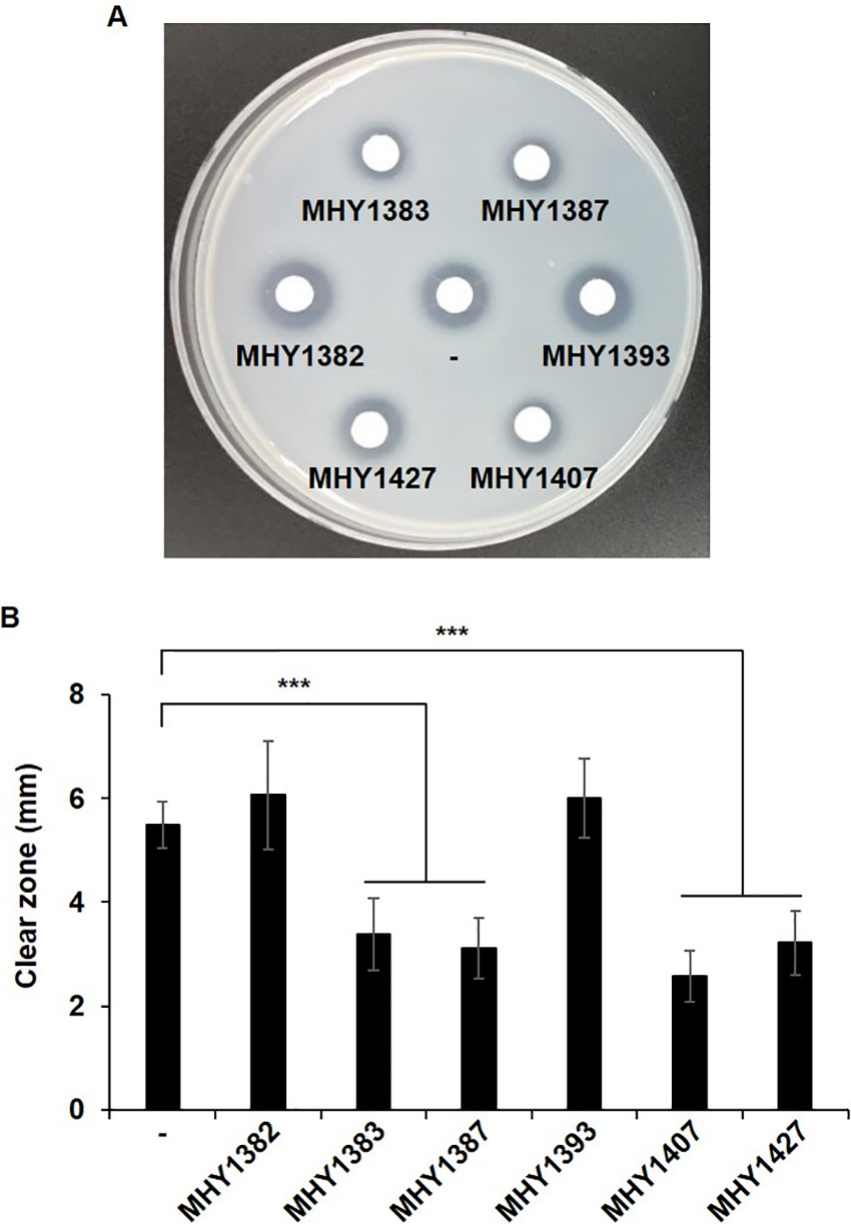
Four MHYs reduced the protease production. Total extracellular protease activity was measured by skim milk plate assay. P. aeruginosa was treated with each MHY (5 μM), and the CSs were applied to the discs on the skim milk plate. (A and B) After a 16-h incubation at 37°C, the clear zones were observed (A), and the average diameters were presented from three independent experiments (B). DMSO was applied as a control (–). ***, *P < *0.005.

In order to know whether these compounds can really suppress the virulence factor production, we measured the total extracellular protease activity when P. aeruginosa cells were treated with each MHY. The result of the skim milk assay showed that the total protease activity was significantly reduced by MHY1383, MHY1387, MHY1407, and MHY1427 (Fig. 2A and B). The molecular structures of these 4 compounds are shown in Fig. S1 in the supplemental material.

**FIG 3:**
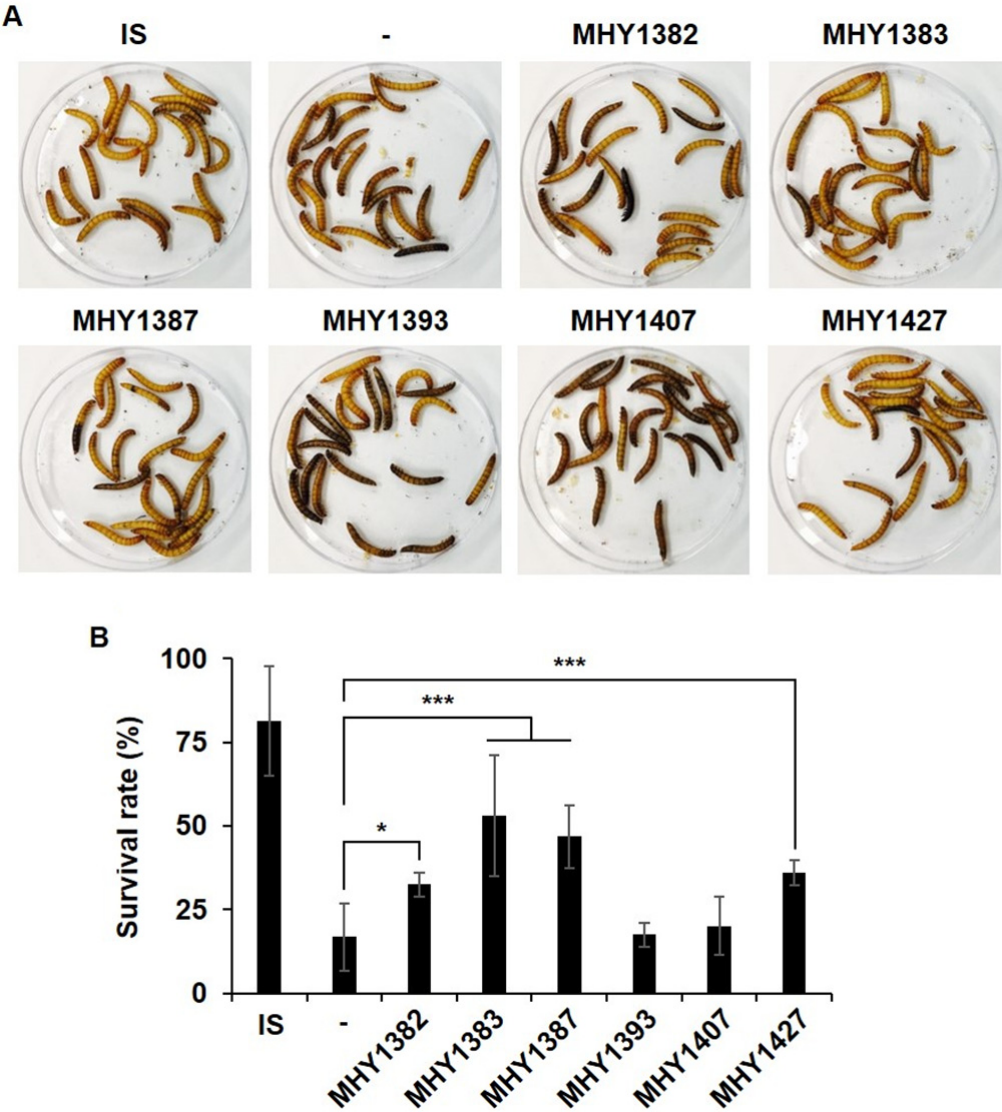
MHY1382, MHY1383, MHY1387, and MHY1427 significantly reduced the toxicity of P. aeruginosa culture supernatant. P. aeruginosa was treated with 5 μM MHYs, and the virulence was measured using a *T. molitor* larval infection assay. (A and B) The melanization and survival were observed at day 2 after injection (A), and the survival rate was calculated from at least three independent experiments (B). Insect saline (IS) was injected as a control, and DMSO (–) was added to P. aeruginosa as an untreated control. *, *P < *0.05; ***, *P < *0.005.

We investigated whether these compounds were able to reduce the toxic effect of P. aeruginosa by using a Tenebrio molitor infection assay. This method was recently developed for the host model system to study bacterial virulence (11, 19). Our result showed that the toxic effect of P. aeruginosa culture supernatant was significantly reduced by MHY1382, MHY1383, MHY1387, and MHY1427 (Fig. 3A and B). However, MHY1407, which reduced the protease production, failed to reduce the toxicity, whereas MHY1382 did not reduce the protease production but reduced the toxicity of P. aeruginosa culture supernatant significantly (Fig. 3A and B). Nevertheless, 3 compounds, MHY1383, MHY1387, and MHY1427, were selected to reduce the P. aeruginosa virulence factors by inhibiting QS.

### MHY1383 and MHY1387 inhibited biofilm formation by reducing intracellular c-di-GMP levels.

**FIG 4:**
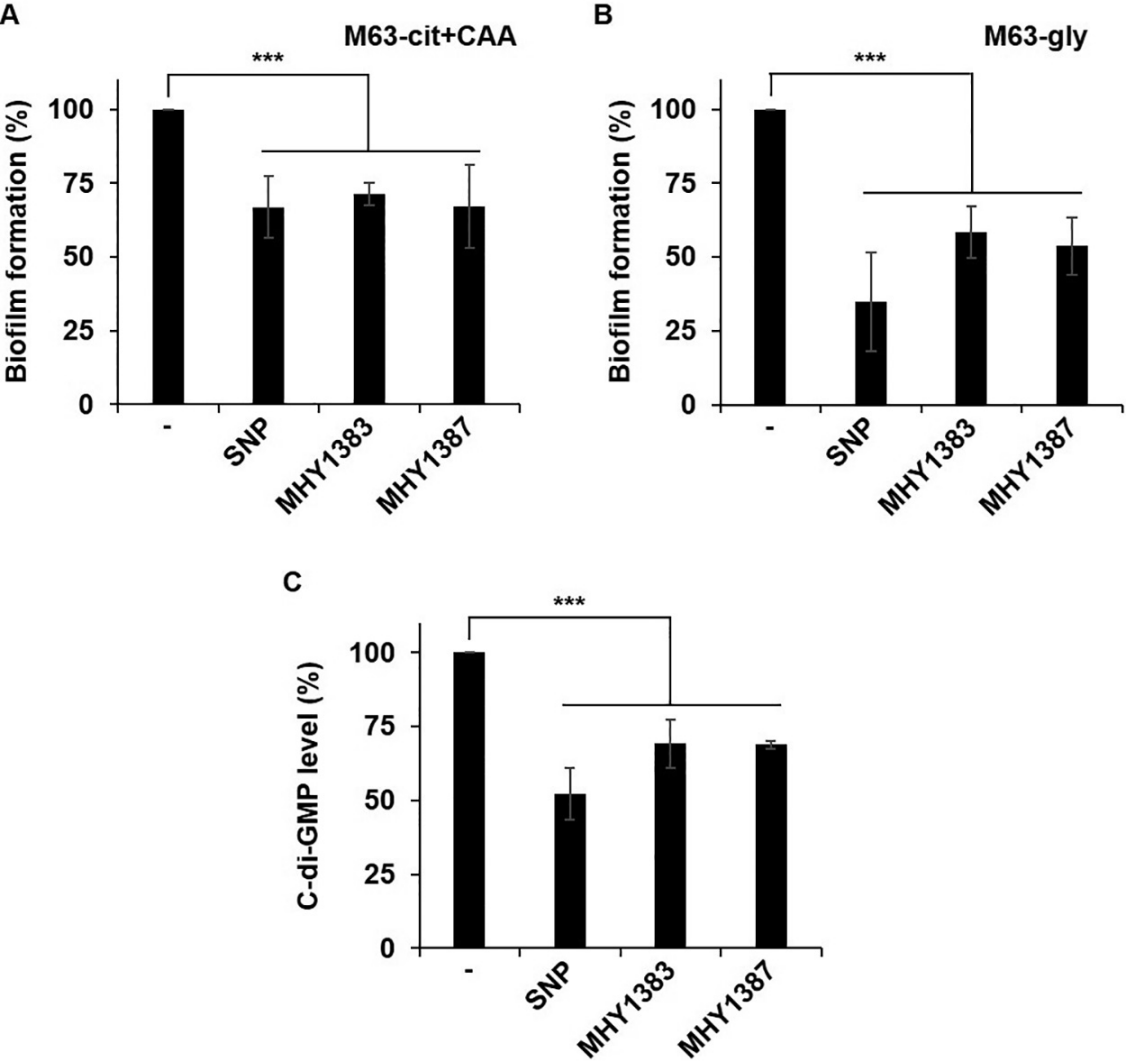
MHY1383 and MHY1387 inhibited the biofilm formation by reducing the intracellular c-di-GMP level. Biofilm formation of wild-type P. aeruginosa was measured by static biofilm assay. (A and B) Biofilms were grown on M63 minimal medium supplemented with 0.2% citrate and 0.5% CAA (M63-cit+CAA) (A) or 0.2% glycerol (M63-gly) (B). (C) P. aeruginosa cells harboring pSKcdrA (*cdrA*_p_-*lacZ* fusion) were grown in LB containing inhibitors for 7 h, and β-galactosidase activity was measured, which reflects the intracellular c-di-GMP level. Data were presented relative to the sample without an inhibitor (which corresponds to 100%). SNP and MHYs were added at 5 μM and 10 μM, respectively. –, no treatment; ***, *P < *0.005.

Next, we investigated the inhibitory effect of MHY1383, MHY1387, and MHY1427 on biofilm formation, another important pathogenic determinant of P. aeruginosa. The static biofilm assay was carried out in two different carbon sources of media, because the biofilm formation of P. aeruginosa is often affected by nutrients (20). Our results showed that 10 μM MHY1383 and MHY1387 significantly inhibited the biofilm formation by 29% and 33%, respectively, in M63-cit+CAA medium and by 42% and 46%, respectively, in M63-gly medium (Fig. 4A and B). However, MHY1427 did not inhibit biofilm formation (Fig. S2).

Sodium nitroprusside (SNP), a nitric oxide (NO)-generating agent, is a well-known biofilm inhibitor (21). When 5 μM SNP was applied for comparison of the inhibition efficacy, it showed a 33% reduction in biofilm formation in M63-cit+CAA medium and 65% reduction in M63-gly medium (Fig. 4A and B). While SNP showed a big difference in the biofilm inhibitory effect between the two types of medium, MHY1383 and MHY1387 showed a small difference. Either way, both showed that biofilm formation was better inhibited when glycerol was given as a carbon source than when citrate + CAA was given.

To address how MHY1383 and MHY1387 inhibit biofilm formation, we investigated the level of an intracellular signaling molecule, c-di-GMP, which plays an important role in controlling biofilm formation in many Gram-negative bacteria (20–22). *cdrA* is strictly regulated by FleQ in response to intracellular c-di-GMP levels (22), so *cdrA*_p_*-lacZ* has been used as a reporter to quantify c-di-GMP and the reliability has been confirmed (21–24). When we treated the cells harboring *cdrA*_p_*-lacZ* with MHYs, the cells treated with MHY1383 and MHY1387 showed a decrease of c-di-GMP levels by approximately 31% (Fig. 4C). Since SNP has been reported to decrease the intracellular c-di-GMP level (21), we treated the reporter cells with 5 μM SNP under the same conditions for comparison, which showed a 47% reduction of c-di-GMP (Fig. 4C). These results were quantitively correlated with the inhibition of biofilm formation by these inhibitors. Therefore, we concluded that, like SNP, both MHY1383 and MHY1387 inhibit biofilm formation by reducing intracellular c-di-GMP. We note that MHY1383 and MHY1387 had no effect on the bacterial growth at 10 μM (Fig. S3A).

### MHY1383 and MHY1387 had effects at very low concentrations.

**FIG 5:**
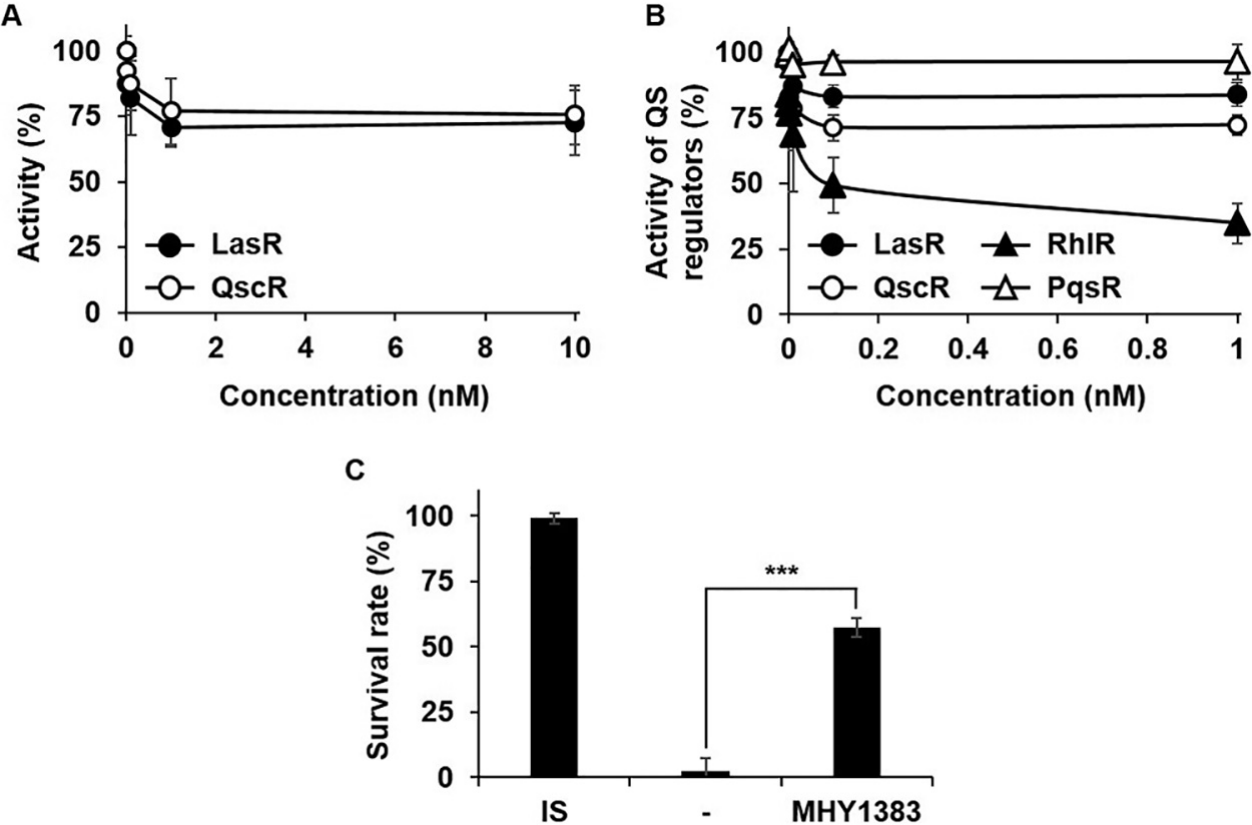
MHY1383 had anti-QS effects at very low concentrations. (A to C) The inhibitory effects of MHY1383 on the activities of QS regulators in E. coli (A) or in P. aeruginosa (B) and the virulence of P. aeruginosa (C) were measured at very low concentrations. (A) the E. coli dual plasmid reporter strains harboring pSC11 (*lasI*_p_-*lacZ*) and pJN105L or pJL101 (*PA1897*_p_-*lacZ*) and pJN105Q were used in the same manner as in Fig. 1. For the QscR activity assay, 3OC12-HSL was added at 100 nM. (B) The reporter plasmids, pSC11, pJL101, pJL501 (*rhlA*_p_-*lacZ*), and pJL301 (*pqsA*_p_-*lacZ*), were introduced into P. aeruginosa, and these reporter strains were treated with MHY1383 at low concentrations. The activities of LasR, QscR, RhlR, and PqsR were measured by β-galactosidase activity assay and presented relative to the untreated sample (which corresponds to 100%). (C) The virulence of P. aeruginosa was measured as in Fig. 3 with the MHY1383 treatment at 100 pM. The survival rate was calculated from at least three independent experiments. ***, *P < *0.005.

Although MHY1383 and MHY1387 seemed to have a somewhat weaker biofilm inhibitory effect than SNP, when the minimum effective concentration was investigated, quite surprising results were obtained. We measured the antivirulence effect of MHY1383 in a lower concentration range and found that MHY1383 began to inhibit the LasR activity at concentrations lower than 1 nM, and the inhibition plateaued at concentrations higher than 1 nM (Fig. 5A). In order to confirm this result, we investigated the inhibitory effect of MHY1383 on QscR, another 3OC12-HSL receptor, using the E. coli dual plasmid reporter harboring pJN101 (*PA1897*_p_-*lacZ*) and pJN105Q. In this experiment, 3OC12-HSL was added at 100 nM, the concentration for the half-maximal activation of QscR (9). Similar to the result with LasR, MHY1383 began to inhibit QscR at concentrations lower than 1 nM and plateaued at concentrations higher than 1 nM (Fig. 5A). In order to confirm whether MHY1383 can inhibit QS activity in P. aeruginosa at such a low concentration, we measured the activities of the main QS regulators, LasR, QscR, RhlR, and PqsR, in P. aeruginosa using their specific promoter-*lacZ* fusions (*lasI*_p_-, *PA1897*_p_-, *rhlA*_p_-, and *pqsA*_p_-*lacZ*). When we treated the P. aeruginosa cells harboring each reporter plasmid with MHY1383, MHY1383 began to inhibit LasR, QscR, and RhlR at concentrations lower than 100 pM and plateaued at concentrations higher than 100 pM (Fig. 5B). Interestingly, RhlR was strongly inhibited, whereas PqsR was not inhibited at all (Fig. 5B), even at a high concentration (10 μM, data not shown). We further confirmed that MHY1383 was also able to significantly reduce the toxicity of P. aeruginosa culture supernatant at 100 pM (Fig. 5C). This result suggests that MHY1383 has the advantage of exerting an effect at very low concentrations.

**FIG 6:**
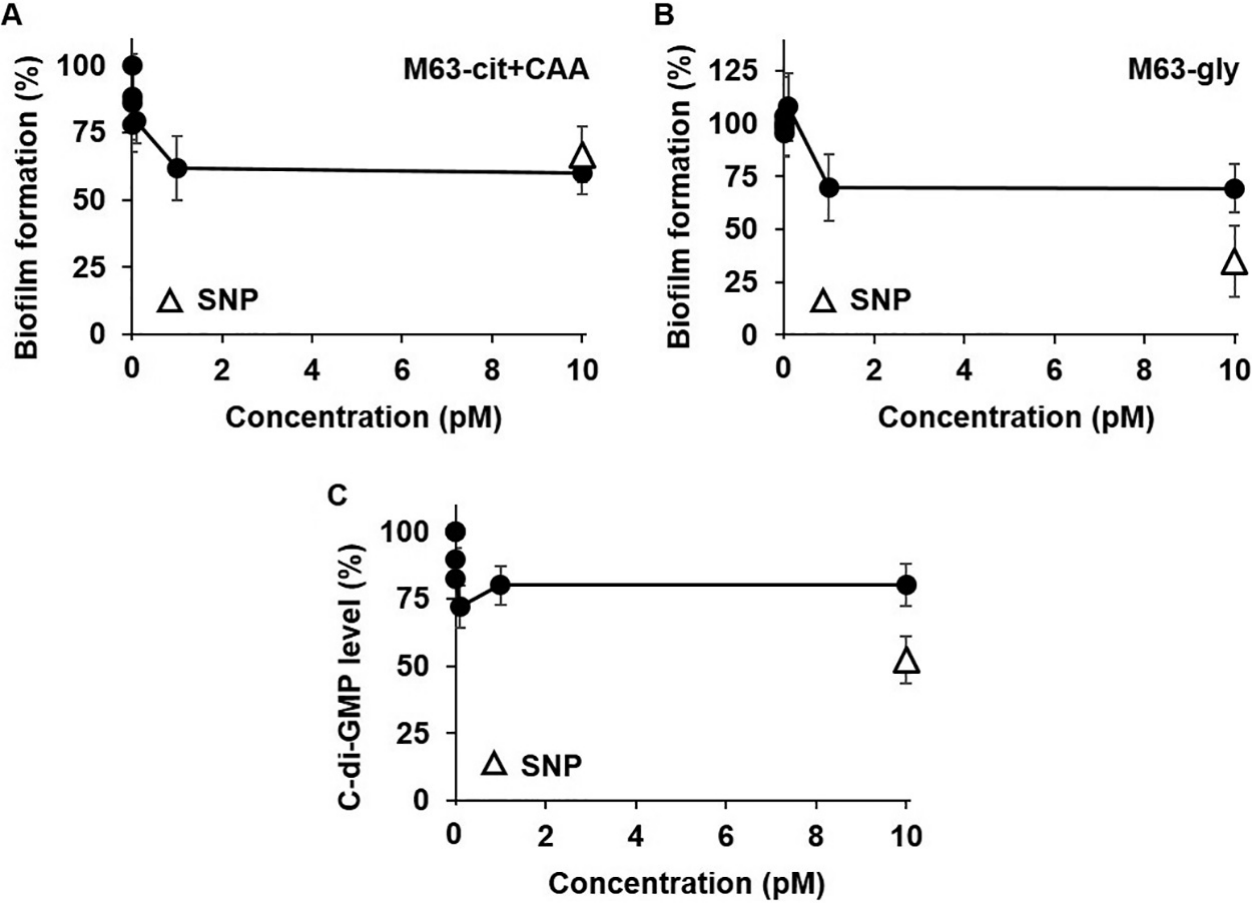
MHY1383 had antibiofilm effects at very low concentrations. (A to C) The inhibitory effects of MHY1383 on biofilm formations in M63-cit+CAA (A) or M63-gly media (B) and the intracellular c-di-GMP levels (C) were measured at very low concentrations. In panels A and B, as in Fig. 4A and B, biofilm formation was measured by static biofilm assay in M63-cit+CAA or M63-gly media. (C) The intracellular c-di-GMP levels were measured as in Fig. 4C. For comparison, the degree of inhibition by 5 μM SNP is indicated as open triangles in panels.

In order to find out whether biofilm formation can be inhibited at low concentrations, the biofilm formation was measured with the treatment of MHY1383 in a low concentration range. As shown in Fig. 6A and B, the biofilm formation was almost maximally inhibited by 1 pM MHY1383 in both M63-cit+CAA and M63-gly. At the concentrations higher than 1 pM, the inhibition plateaued. The intracellular c-di-GMP level was maximally reduced at 0.1 pM MHY1383 and also plateaued at concentrations higher than 0.1 pM (Fig. 6C).

**FIG 7:**
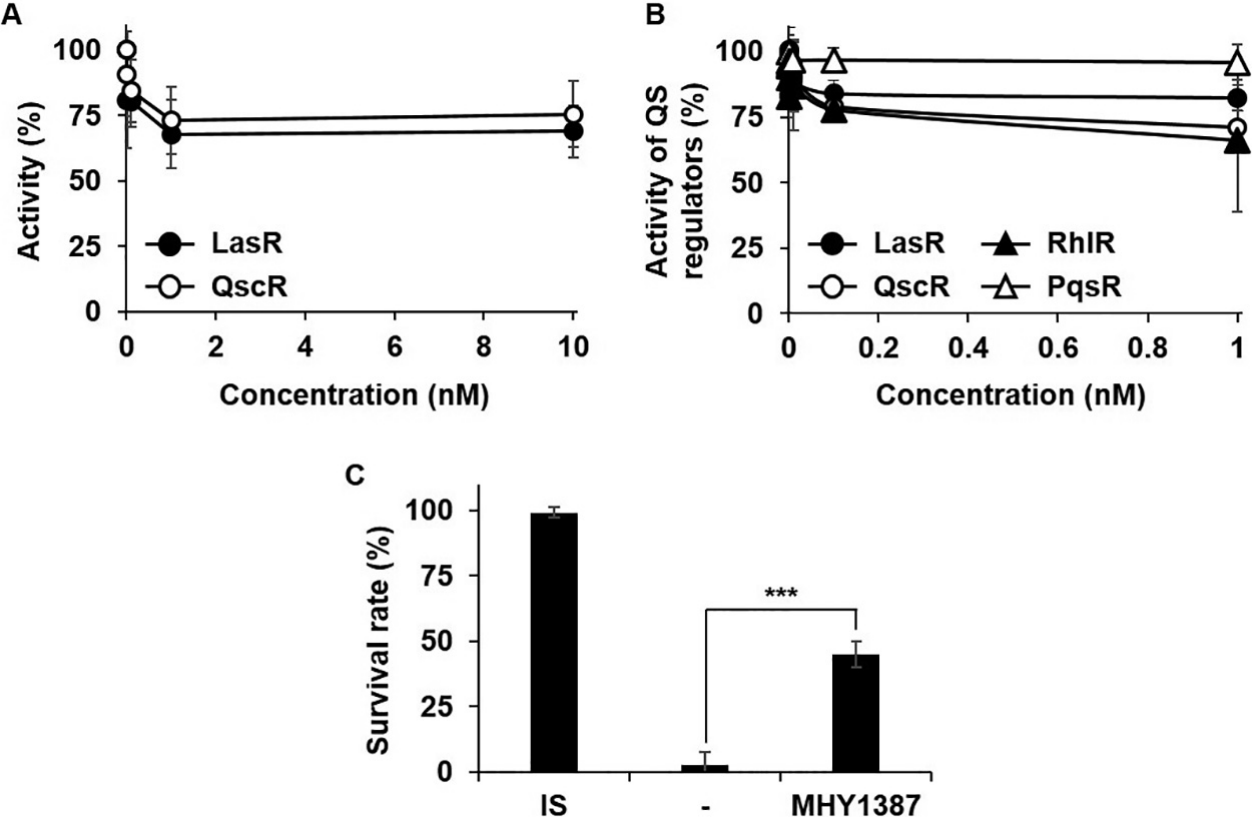
MHY1387 also effectively inhibited QS at very low concentrations. (A to C) The inhibitory effects of MHY1387 on QS activities in E. coli (A) or in P. aeruginosa (B) and virulence of P. aeruginosa (C) were measured at very low concentrations. All experiments were performed in the same manner as in Fig. 5.

We performed the same experiment with another candidate, MHY1387. MHY1387 also began to inhibit the activity of LasR and QscR in an E. coli system below 1 nM and plateaued above 1 nM (Fig. 7A). When we measured the activities of the four different QS regulators in the P. aeruginosa cells, the MHY1387 treatment also inhibited LasR, QscR, and RhlR at concentrations lower than 100 pM and plateaued at concentrations higher than 100 pM, whereas PqsR was not inhibited (Fig. 7B), even at a high concentration (10 μM, data not shown). Unlike MHY1383, MHY1387 inhibited RhlR to a similar extent as QscR (Fig. 7B). MHY1387 was also able to significantly reduce the toxicity of P. aeruginosa culture supernatant at 100 pM (Fig. 7C).

**FIG 8:**
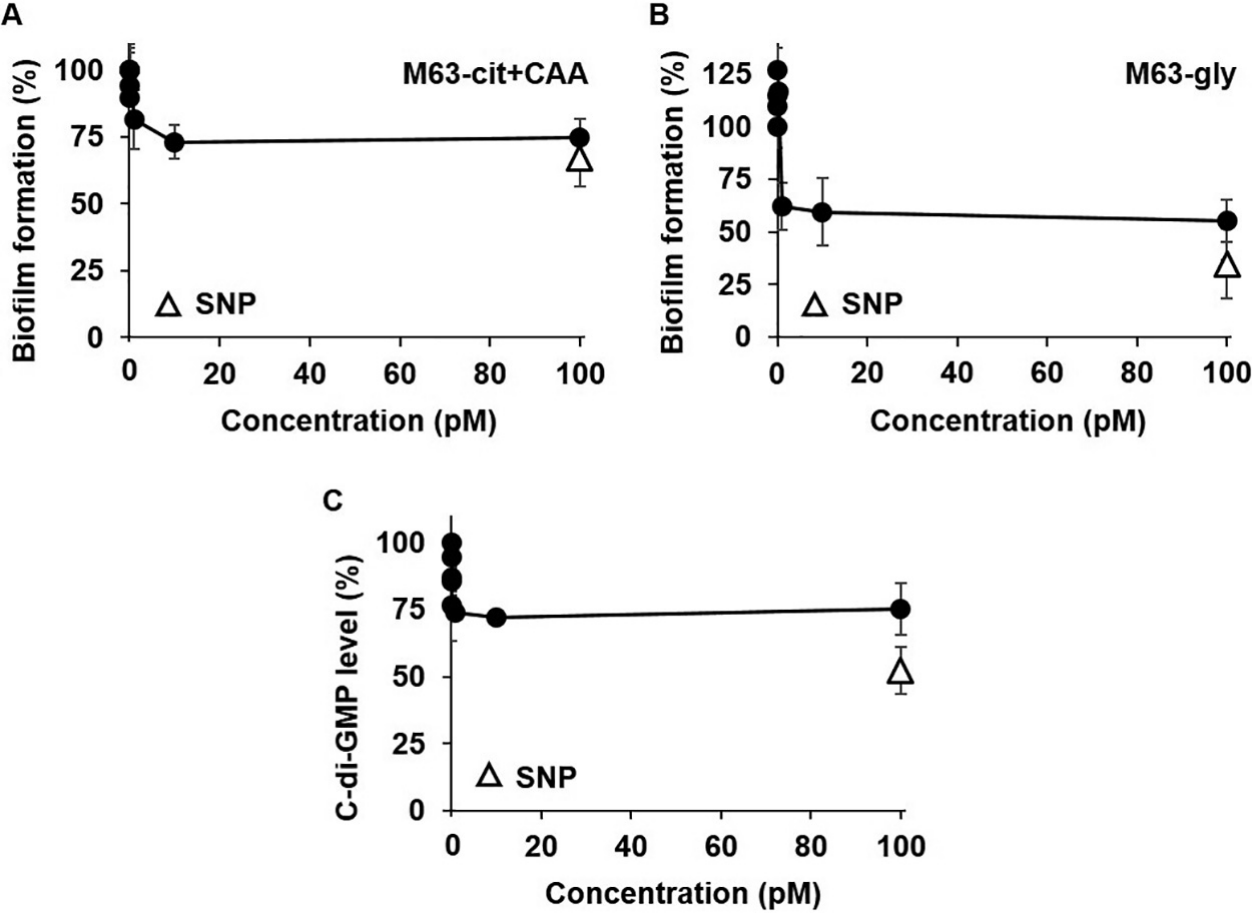
MHY1387 also effectively inhibited biofilm formation at very low concentrations. The inhibitory effects of MHY1387 on biofilm formation in M63-cit+CAA (A) or M63-gly media (B) and the intracellular c-di-GMP levels (C) were measured at very low concentrations. All experiments were performed in the same manner as in Fig. 6, and the degree of inhibition by 5 μM SNP was also indicated as open triangles for comparison.

The biofilm formation was maximally inhibited by 10 pM MHY1387 in M63-cit+CAA (Fig. 8A), while in M63-gly medium, MHY1387 inhibited biofilm formation at 1 pM (Fig, B). MHY1387 reduced the intracellular c-di-GMP level at 1 pM (Fig. 8C). At higher concentrations, the biofilm inhibition and reduction of c-di-GMP all plateaued similarly to the MHY1383 treatment. We note that MHY1383 and MHY1387 also had no effect on the bacterial growth in M63-cit+CAA and M63-gly media (Fig. S3B and C).

Summarizing these results, both MHY1383 and MHY1387 inhibited QS and virulence factor production at a very low concentration of about 100 pM to a similar degree. Both MHY1383 and MHY1387 showed slightly weaker levels of biofilm inhibition compared to SNP, but the effective concentration was 10^6^ times lower than that of SNP. In particular, MHY1383 inhibited biofilm formation at a lower concentration than MHY1387.

## DISCUSSION

In this study, we screened a series of synthetic compounds and finally found that two compounds, MHY1383 and MHY1387, have both antivirulence and antibiofilm activities. These two compounds have been previously reported for different activities as well as their synthesis methods; MHY1383, azo-resveratrol (Fig. S1), inhibited mushroom tyrosinase (25), while MHY1387, 5-[4-hydroxy-3,5-methoxybenzy]-2-thioxodihydropyrimidine-4,6[1H,5H]-dione (Fig. S1), inhibited the production of reactive oxygen species (ROS) and peroxynitrate *in vitro*, therefore suppressing lipopolysaccharide (LPS)-induced oxidative stress and NF-κB activation in the mouse liver when orally administered (26). It is currently unclear whether these activities are related to the anti-QS and antibiofilm activities against bacteria we found in this study. Regardless, this is the first report that these two MHYs have an effect of inhibiting QS and biofilm formation of bacteria.

The advantages of these two MHYs can be summarized as follows. First, they have both antivirulence and antibiofilm effects. Second, they work at extremely low concentrations. Third, they have no effect on growth. The first advantage is important because pathogens can avoid the antibiotic medication by appropriately changing their infection mode between acute and chronic. Therefore, a substance capable of inhibiting both virulence factor production and biofilm formation at the same time would have a much higher infection control potential.

The second advantage is also very important. These two compounds showed an effect at extremely low concentrations, and in particular, they inhibited biofilm formation in the range of 1 to 10 pM. Well-known biofilm inhibitors, SNP and anthranilate, inhibited biofilm at 5 μM and 100 μM, respectively (21, 24). Our two MHYs showed similar effects at concentrations 10^6^ or 10^8^ times lower than these. The anti-QS effect of these two MHYs was also maximized at around 100 pM, which is much lower than the concentration of the previously screened anti-QS substances that were usually effective at concentrations higher than 0.1 μM (15, 16, 27, 28). At first glance, MHY1383 and MHY1387 appear to have weak anti-QS effects, but our results show that they can actually reduce the toxicity of P. aeruginosa virulence factors in a small-animal model (Fig. 3, 5C, and 7C).

Third, in contrast to conventional antibiotics, these MHYs do not interfere with bacterial growth or viability. Therefore, cells are not expected to generate antibiotic resistance, because they are not under selective pressure. Of course, this is merely conceptual, and we do not say that resistance will never occur, but we believe that it has logical validity that these compounds can reduce the possibility of resistance development (18). It is possible to inhibit virulence of P. aeruginosa without killing cells; such antipathogenic drugs may be used in combination with antibiotics. All of these advantages demonstrate that these MHYs are good drug candidates to prevent P. aeruginosa infection.

Our results strongly suggest that both MHY1383 and MHY1387 act in a noncompetitive manner against the acyl-HSL or the target involved in biofilm formation that has not yet been identified. This suggestion is supported by the facts that they worked at a much lower concentration than the added 3OC12-HSL and that the effect did not improve even when their concentrations increased. This also indicates that MHY1383 and MHY1387 are distinct from other previously known QS inhibitors or biofilm inhibitors, which means that the range of materials we can choose for a new antibiotic strategy has been expanded.

## MATERIALS AND METHODS

### Bacterial strains, culture conditions, and plasmids.

The organisms and plasmids used in this study are listed in Table S1. PAO1, a P. aeruginosa wild-type strain, was used in most experiments. For the E. coli dual plasmid reporter system, the E. coli DH5α strain was used. These bacterial strains were mostly grown in Luria-Bertani medium (LB; 10 g/liter tryptone, 5 g/liter yeast extract, and 5 g/liter NaCl) at 37°C with vigorous shaking at 170 rpm. Growth was measured by optical density at 600 nm (OD_600_). For solid media, agar was added at 1.5% (wt/vol). l-arabinose (0.4%) was used to induce protein expression. Antibiotics were used at the following concentrations: carbenicillin, 150 μg/ml; ampicillin, 100 μg/ml; gentamicin, 10 μg/ml.

### Chemicals for inhibitor screening.

An in-house synthetic chemical library (MHYs) was used for screening antivirulence activity against P. aeruginosa. This library contained 20 hydroxamic and carboxylic acid derivatives, 13 bis-aryl derivatives, 23 benzothiazoles/benzimidazoles, 13 azo/diazo compounds, 8 nonclassical nucleosides, 2 flavonoids, 30 benzylidene derivatives, and 17 other derivatives (Fig. S4). These compounds were dissolved in dimethyl sulfoxide (DMSO) and diluted to the indicated concentrations in the media.

### Measurement of QS inhibition activity.

The activities of QS regulators were measured using the regulator-specific promoter-*lacZ* fusion plasmids, pSC11 (*lasI*_p_-*lacZ*, for the LasR activity), pJL101 (*PA1897*_p_-*lacZ* for the QscR activity), pJL501 (*rhlA*_p_-*lacZ* for the RhlR activity), and pJL301 (*pqsA*_p_-*lacZ* for the PqsR activity). In the E. coli system analysis, the dual plasmid-based LacZ bioassay was used to measure the QS inhibition activity of MHYs, as described previously (9, 16). Two compatible plasmids, pSC11 and pJN105L or pJL101 and pJN105Q, were cotransformed into E. coli DH5α to make the reporter strains. pJN105L and pJN105Q are the LasR and QscR expression plasmids, respectively (9), while pSC11 and pJL101 are the *lasI*_p_-*lacZ* and *PA1897*_p_-*lacZ* fusion plasmids, respectively (9, 29). The E. coli reporter strain that harbors pSC11 and pJN105L reflects the LasR activity, and the strain that harbors pJL101 and pJN105Q reflects the QscR activity. Hence, they can be used for measuring the activities of LasR and QscR in response to 3OC12-HSL. The transformant E. coli was cultured up to 0.3 of OD_600_ in LB broth containing gentamicin and ampicillin at 37°C with vigorous shaking. Then, arabinose (0.4%), 3OC12-HSL (50 nM for LasR activation and 100 nM for QscR activation), and MHYs (concentrations are indicated in the figures) were added to the culture at the indicated concentrations. After 90-min of further cultivation, β-galactosidase activity was measured as described below. In P. aeruginosa, the reporter plasmids were transformed into PAO1. The overnight cultures of each reporter strain were inoculated at an OD_600_ of 0.02 into fresh LB broth containing carbenicillin and cultivated with MHYs for 7 h, and the β-galactosidase activity was measured as described below. Dimethyl sulfoxide (DMSO), the solvent for MHYs, was diluted in the same manner as the MHYs and added as a control.

### β-Galactosidase activity assay.

β-Galactosidase activity was measured using the Galacto-Light Plus kit (Applied Biosystems, USA), as described elsewhere (9). Aliquots of cultures were briefly measured for the OD_600_ and mixed with 10% of chloroform. After vigorous vortexing and a 15-min incubation at room temperature, 10 μl of supernatant was transferred to new tubes, and substrate solution was added. After a 1-h incubation in the dark at room temperature, 150 μl of light emission solution (Accelerator II) was added, and luminescence was promptly measured using a multiwell plate reader (Tristar LB941; Berthold). Final β-galactosidase activities were obtained by normalizing the luminescence by OD_600_ (luminescence/OD_600_) and presented as a relative value to those with no inhibitor.

### Measurement of total protease activity.

P. aeruginosa cells were grown overnight in LB, diluted 1:100 into fresh 5 ml of fresh LB containing MHYs, and cultivated with vigorous shaking at 37°C up to an OD_600_ of 2.0. Cells were removed by centrifugation at 4°C, and the culture supernatants (CSs) were taken to be filtered through a 0.2-μm filter (GVS Abluo syringe filter). To measure the total protease activity, 5 μl of CSs were dropped onto the discs on skim milk agar plates (0.5% skim milk, 0.5% peptone, 0.1% glucose, and 1.5% agar) and incubated overnight at 37°C. The total protease activity was then estimated with the diameter of the clear zones. DMSO was diluted in the same manner as the MHYs and applied as a control.

### Virulence analysis.

The CSs prepared as described above were concentrated 10 times in 10 kDa cutoff Centricon (Vivaspin; Satorious), 5 μl of which was injected into *Tenebrio molitor* larvae using a microsyringe. As a control, the same volume of insect saline (IS; 130 mM NaCl, 5 mM KCl, and 1 mM CaCl_2_) was injected. The larvae were incubated in petri dishes at 25°C for 2 days, and the surviving larvae were counted to calculate the survival rate.

### Measurement of biofilm formation.

Biofilm formation was quantified using a static biofilm assay, as described previously with slight modification (30). The overnight cultures of cells were 2%-inoculated into fresh medium on a 96-well polystyrene plate. The medium was M63 minimal medium (M63 salt [KH_2_PO_4_, 3 g/liter; K_2_HPO_4_, 7 g/liter; (NH_4_)_2_SO_4_, 2 g/liter], 1 mM MgSO_4_) containing either of two different carbon sources, 0.5% casamino acid (CAA)+0.2% citrate (M63-cit+CAA), or 0.2% glycerol (M63-gly). MHYs were added at the indicated concentrations into this medium, and the cells were grown at 37°C for 24 h without shaking. After the cell growth was measured by OD_600_, planktonic cells were poured out, and the plate was washed with water and dried for 10 min. Then, 180 μl of crystal violet (0.1% wt/vol) was added to each well and incubated for 10 min to stain the biofilms attached to the well surface. After a brief wash, the biofilm-staining crystal violet was dissolved in 200 μl of 30% acetic acid. The biofilm formation was quantified from the staining levels measured by absorbance at 600 nm (A_600_), which was normalized by cell growth (OD_600_). Data were presented relative to the sample without an inhibitor (which corresponds to 100%).

### Measurement of intracellular c-di-GMP.

The intracellular c-di-GMP levels were measured using a *cdrA*_p_-*lacZ* reporter as described previously (23, 24). A P. aeruginosa strain harboring pSKcdrA was grown overnight, 1%-diluted into fresh LB containing MHYs, cultivated for 7 h, and then measured for β-galactosidase activity, which reflects the intracellular c-di-GMP level. For comparison, 5 μM sodium nitroprusside (SNP; Bio Basic, Inc., Canada) was added under the same conditions. Data were presented relative to the sample without an inhibitor (which corresponds to 100%).

### Statistical analysis.

Student’s *t* test (two-sample assuming equal variances) was used to determine the significance of differences using Microsoft Office Excel. *P < *0.05 was considered significant. All experiments were carried out in triplicate and repeated at least twice independently.
