# Supplementary material for: Antipathogenic Compounds That Are Effective at Very Low Concentrations and Have Both Antibiofilm and Antivirulence Effects against Pseudomonas aeruginosa
Source: Microbiol Spectr. 2021 Sep 8;9(2):e00249-21. doi: 10.1128/Spectrum.00249-21 (PMC8557914; doi:10.1128/Spectrum.00249-21)
Supplement: SUPPLEMENTAL FILE 1 — Supplemental material. Download SPECTRUM00249-21_Supp_1_seq13.pdf, PDF file, 0.8 MB [file spectrum00249-21_supp_1_seq13.pdf]

Anti-pathogenic compounds that are effective at very low concentrations and have both anti-biofilm and anti-virulence effects against *Pseudomonas aeruginosa*

Hyeon-Ji Hwang[1], Heejeong Choi[2], Sojeong Hong[2], Hyung Ryong Moon[2], and Joon-Hee Lee[1*]

[1]Department of Pharmacy, College of Pharmacy, Pusan National University, Busan, 46241, South Korea

[2]Department of Manufacturing Pharmacy, College of Pharmacy, Pusan National University, Busan, 46241, South Korea

Table S1. Bacterial strains and plasmids used in this study.

Name	Genotype	
P. aeruginosa		
PAO1	Wild type *P. aeruginosa*	(1)
E. coli		
DH5α	*supE44 ΔlacU169* (*ϕ80 lacZΔM15*) *hsdR17 recA1 endA1 gyrA96 thi-1 relA1*	Lab. collection
**Small animals**		
Tenebrio molitor	Yellow mealworm	Lab. collection
**Plasmids**		
pSC11	$lasI_{P}$-*lacZ* reporter in pQF50, Ap^R	(2)
pJL101	$PA1897_{P}$-*lacZ* reporter in pQF50, Ap^R	(3)
pJL501[*]	$rhlA_{P}$-*lacZ* reporter in pQF50, Ap^R	This study
pJL301	$pqsA_{P}$-*lacZ* reporter in pQF50, Ap^R	(4)
pJN105L	*lasR* orf in pJN105, Gm^R	(3)
pJN105Q	*qscR* orf in pJN105, Gm^R	(3)
pSKcdrA	$cdrA_{P}$-*lacZ* reporter in pQF50, Ap^R	(5)

Ap^R, ampicillin and carbenicillin resistance; Gm^R, gentamicin-resistance; orf, open reading frame.

[*]This $rhlA_{P}$-*lacZ* fusion was constructed by cloning the PCR-amplifying *rhlA* promoter region (-486 ~ +12 relative to start codon) into the *Sma*I-site of pQF50.

Fig. S1.

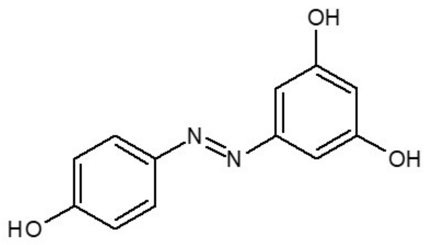

MHY1383

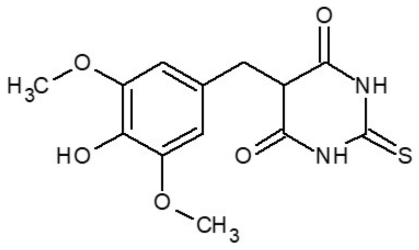

MHY1387

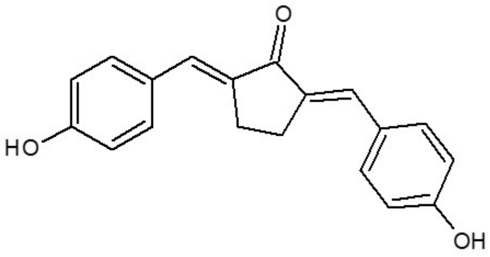

MHY1407

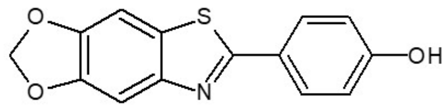

MHY1427

Fig. S1. The molecular structure of MHY1383, MHY1387, MHY1407, and MHY1427. These 4 compounds were selected from the primary screening for the inhibitory effects on QS and protease production.

Fig. S2.

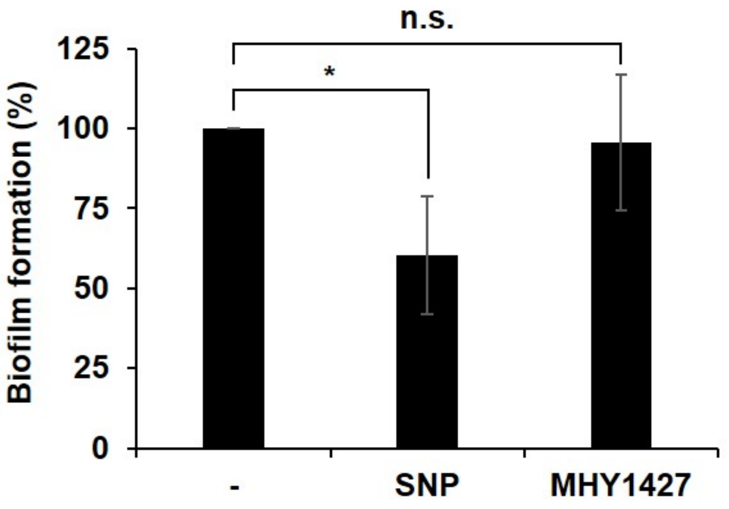

Fig. S2. MHY1427 had no effect on biofilm formation. Biofilm formation of *P. aeruginosa* was measured with 10 μM MHY1427 treatment by static biofilm assay in M63-cit+CAA medium. The inhibitory effect of 5 μM SNP was presented for comparison. Data were relatively presented to the sample without an inhibitor (which corresponds to 100%). -, no treatment; n.s., not significant; *, $p < 0.05$.

Fig. S3.

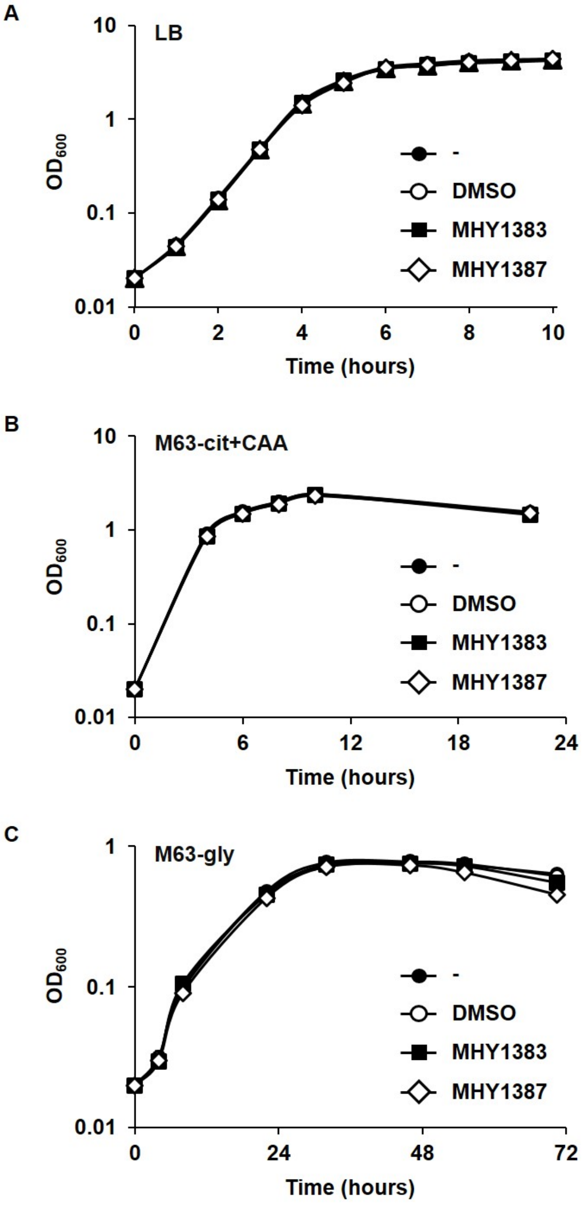

Fig. S3. MHY1383 and MHY1387 had no effect on the growth of *P. aeruginosa*. The growth curves of *P. aeruginosa* were monitored in LB (A), M63-cit+CAA (B), or M63-gly (C) media containing 10 μM MHY1383 and MHY1387 to investigate any potential toxicity. Cells were cultivated at 37°C for 10, 22, and 70.5 hours, respectively and OD_{600} was measured at the indicated hours. The same volume of DMSO was added as a solvent control.

Fig. S4.

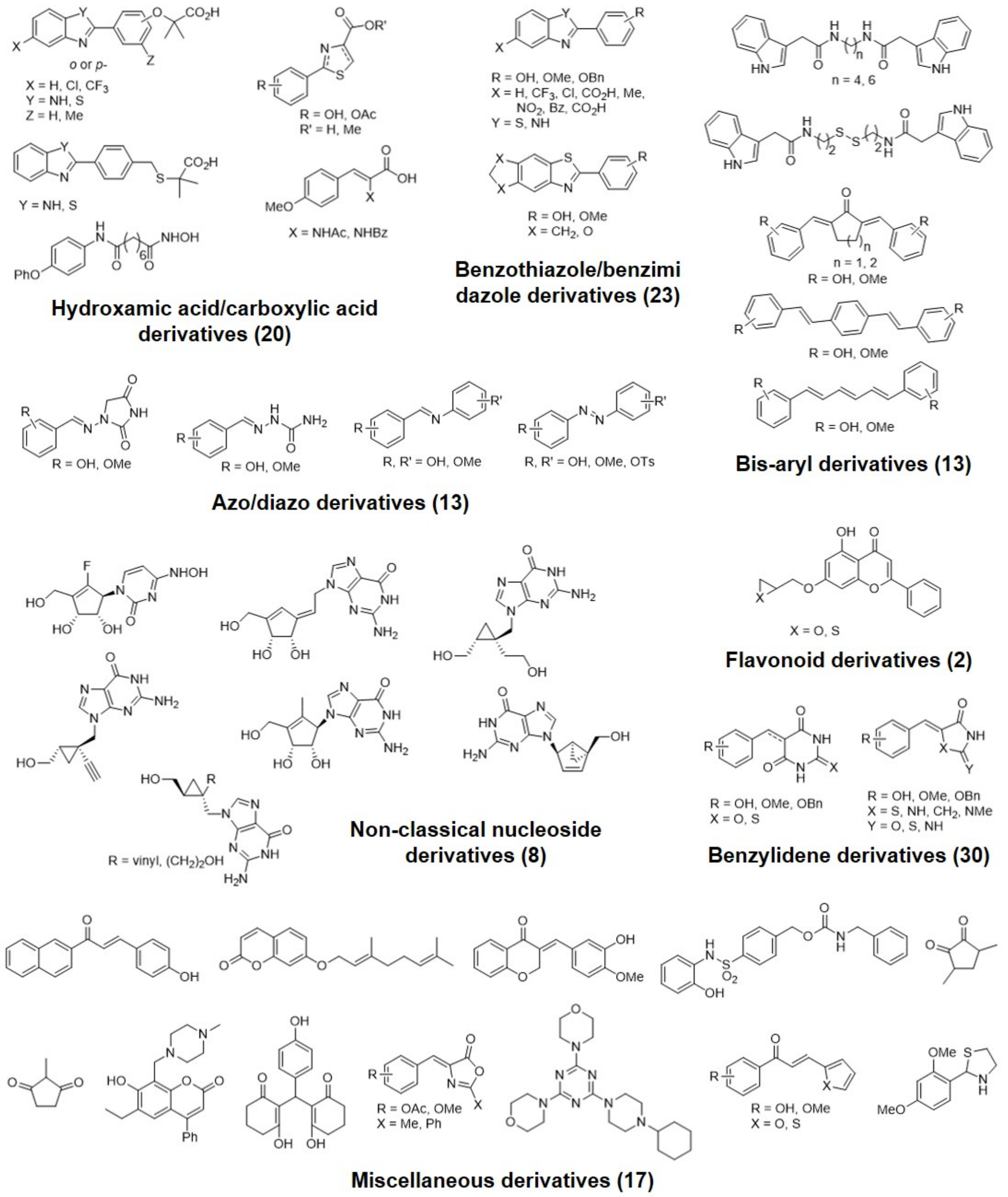

Fig. S4. The chemical library used in this study. The chemicals in this library were named as MHY + serial number. The representative molecular structures of MHYs in each category are shown. The numbers in parentheses refer to the number of compounds tested in each category.

Reference

1. Pearson JP, Pesci EC, Iglewski BH. 1997. Roles of Pseudomonas aeruginosa las and rhl quorum-sensing systems in control of elastase and rhamnolipid biosynthesis genes. J Bacteriol 179:5756-67.
2. Chugani SA, Whiteley M, Lee KM, D'Argenio D, Manoil C, Greenberg EP. 2001. QscR, a modulator of quorum-sensing signal synthesis and virulence in *Pseudomonas aeruginosa*. Proc Natl Acad Sci USA 98:2752-2757.
3. Lee JH, Lequette Y, Greenberg EP. 2006. Activity of purified QscR, a Pseudomonas aeruginosa orphan quorum-sensing transcription factor. Mol Microbiol 59:602-9.
4. Choi Y, Park HY, Park SJ, Kim SK, Ha C, Im SJ, Lee JH. 2011. Growth phase-differential quorum sensing regulation of anthranilate metabolism in Pseudomonas aeruginosa. Mol Cells 32:57-65.
5. Kim SK, Park HY, Lee JH. 2015. Anthranilate deteriorates the structure of Pseudomonas aeruginosa biofilms and antagonizes the biofilm-enhancing indole effect. Appl Environ Microbiol 81:2328-38.
